# Periprocedural Management of Direct Oral Anticoagulants Should Be Guided by Accurate Laboratory Tests

**DOI:** 10.1097/AAP.0000000000000448

**Published:** 2016-09-22

**Authors:** Sarah Lessire, Jonathan Douxfils, Anne-Sophie Dincq, François Mullier

**Affiliations:** Université catholique de Louvain CHU UCL Namur Department of Anesthesiology Namur Thrombosis and Hemostasis Center (NTHC), Namur Research Institute for Life Sciences (NARILIS) Yvoir, Belgium; Université de Namur Department of Pharmacy Namur Thrombosis and Hemostasis Center (NTHC), NARILIS Namur, Belgium; Université catholique de Louvain CHU UCL Namur Department of Anesthesiology Namur Thrombosis and Hemostasis Center (NTHC), NARILIS Yvoir, Belgium; Université catholique de Louvain CHU UCL Namur, Haematology Laboratory, Namur Thrombosis and Hemostasis Center (NTHC), NARILIS Yvoir, Belgium

To the Editor:

We read with interest the recent discussion^1,2^ about the 2015 American Society of Regional Anesthesia and Pain Medicine guidelines concerning interventional spine and pain procedures in patients on direct oral anticoagulants (DOACs).^3^ We agree that the interruption of DOACs should not be based only on their respective half-life but also on the residual drug concentration. Indeed, a recent multicenter study showed a high interindividual variability of DOACs' plasma concentration. Furthermore, a poor correlation between renal function and plasma concentration of rivaroxaban, apixaban, and dabigatran was found, except for dabigatran measurements at trough.^4^ Douketis et al stated recently that stopping dabigatran 48 hours before the procedure in patients with creatinine clearance greater than 50 mL/min may not be long enough to achieve normal coagulation tests in greater than 95% of patients. Indeed, in the prospective study of Douketis et al,^5^ the mass spectrometry (LC-MS/MS) measured a dabigatran level greater than 20 ng/mL in approximately 16% of patients undergoing high bleeding risk procedures.

However, some issues need to be addressed regarding the laboratory assays. Contrary to the statement of Benzon et al,^1^ it should be mentioned that specific tests to measure DOACs are more widely available than in the past years, and some of them are already CE marked and easily available in the European Union (ie, The Hemoclot Thrombin Inhibitor [HTI] and the STA-Ecarin Chromogenic Assay II [ECA-II]). The US Food and Drug Administration is now considering an expansion of the availability of these tests in laboratories in the United States. Regarding the interpretation of the laboratory tests made by Douketis et al in their study, we have the following comments.

First, the activated partial thromboplastin time can be normal in the presence of therapeutic concentrations of dabigatran and is therefore not recommended for the detection of low dabigatran plasma concentration. In addition, activated partial thromboplastin time is not specific to dabigatran and the sensitivity depends on the reagent and the coagulometer.^6^

Second, the thrombin time (TT) shows the highest sensitivity toward dabigatran. In the study of Douketis et al,^5^ TT was higher than the upper limit of the reference range in 43% of patients undergoing a high bleeding risk surgery. This percentage could be different in other laboratories, as TT is not standardized and is affected by many variables (ie, type of thrombin or the clot detection method).^6^ Therefore, it is not possible to draw conclusions from multicenter studies in which centers use different procedures for TT. Thus, a normal TT can only reasonably exclude the presence of clinically relevant concentrations of dabigatran.

Third, concerning the LC-MS/MS, the authors should have mentioned if their measurements include acyl-glucuronides, which can account for 20% of total dabigatran. Nevertheless, this proportion seems to be smaller for low dabigatran concentrations.^6^

Finally, the conventional HTI (a diluted TT) is affected by a limit of quantitation between 30 and 50 ng/mL,^6^ and not 20 ng/mL as reported by the authors.^5^ This limit of quantitation was not able to measure accurately DOAC concentrations encountered in the perioperative setting. Therefore, it is recommended to adapt the calibration and use an appropriate method for the measurements of low DOAC concentrations (ie, using the HTI LOW or the ECA-II for dabigatran) as they were found more accurate than the standard method (HTI).^6^

These laboratory assays are helping physicians to adapt the period of interruption of DOACs to achieve residual plasma concentrations allowing high bleeding procedures. For example, Spyropoulos et al^7^ increased the period of arrest for dabigatran before a high bleeding procedure in patients with creatinine clearance greater than 50 mL/min, from 48 hours in the first prospective study to 72 hours. Thus, despite their attractive pharmacokinetic properties, the high interindividual variability of plasma concentrations observed with DOACs supports further studies with accurate laboratory tests to validate a unique periprocedural management.

**Sarah Lessire, MD** Université catholique de Louvain CHU UCL Namur Department of Anesthesiology Namur Thrombosis and Hemostasis Center (NTHC), Namur Research Institute for Life Sciences (NARILIS) Yvoir, Belgium **Jonathan Douxfils, PharmD, PhD** Université de Namur Department of Pharmacy Namur Thrombosis and Hemostasis Center (NTHC), NARILIS Namur, Belgium **Anne-Sophie Dincq, MD** Université catholique de Louvain CHU UCL Namur Department of Anesthesiology Namur Thrombosis and Hemostasis Center (NTHC), NARILIS Yvoir, Belgium **François Mullier, PharmD, PhD** Université catholique de Louvain CHU UCL Namur, Haematology Laboratory, Namur Thrombosis and Hemostasis Center (NTHC), NARILIS Yvoir, Belgium
